# Priming methods affected deterioration speed of primed rice seeds by regulating reactive oxygen species accumulation, seed respiration and starch degradation

**DOI:** 10.3389/fpls.2023.1267103

**Published:** 2023-10-04

**Authors:** Muyao Ren, Biao Tan, Jiayi Xu, Zhengpeng Yang, Huabin Zheng, Qiyuan Tang, Xiaoli Zhang, Weiqin Wang

**Affiliations:** ^1^ College of Agronomy, Hunan Agricultural University, Changsha, China; ^2^ Rice Research Institute of Guangxi Academy of Agricultural Sciences, Guangxi Key Laboratory of Rice Genetics and Breeding, Nanning, China

**Keywords:** rice, seed priming, seed deterioration, priming methods, reactive oxygen species

## Abstract

**Introduction:**

Seed priming is a pre-sowing seed treatment that is beneficial for rice seed germination and seedling growth, but the reduced seed longevity after seed priming greatly limited its adoption. The deterioration of primed seeds showed large differences among different studies, and the priming method might play an important role in regulating the deterioration speed of primed seeds. However, whether and how the priming method affected the deterioration of primed rice seeds during storage remains unknown.

**Methods:**

In this study, two typical seed priming methods, namely hydropriming (HP) and osmopriming (PEG) were compared under artificially accelerated aging conditions, the changes in germination performance, starch metabolism, seed respiration and reactive oxygen species accumulation before and after accelerated aging were determined.

**Results and discussion:**

Hydroprimed rice seeds exhibited significantly faster deterioration speed than that of PEG-primed seeds in terms of germination speed and percentage. Meanwhile, α-amylase activity and total soluble sugar content in hydroprimed seeds were reduced by 19.3% and 10.0% respectively after aging, as compared with PEG-primed seeds. Such effects were strongly associated with the increased reactive oxygen generation and lipid peroxidation, as the content of superoxide anion, hydrogen peroxide and malondialdehyde in hydroprimed seeds were 4.4%, 12.3% and 13.7% higher than those in PEG-primed seeds after aging, such effect could be attributed to the increased respiratory metabolism in hydroprimed seeds. In addition, the simultaneous use of N-acetylcysteine with HP and PEG priming greatly inhibited the deterioration of primed rice seeds, suggesting that the ability to scavenge reactive oxygen species may be the key factor affecting the speed of deterioration in primed rice seeds during storage.

## 1 Introduction

Seed priming is a pre-sowing seed treatment that has been widely used by farmers and seed companies to improve seed germination performance (Paparella et al., 2015). The basic procedures of seed priming include the controlled seed hydration to induce various metabolic processes during the early phase of germination, and the drying of primed seeds to an initial moisture content before radicle emergence (Heydecker and Gibbins, 1978). In recent years, the effects of seed priming have been intensively studied in rice, maize, lettuce, tomato and other crops and the benefits of seed priming to increase seed germination, uniformity of seedling emergence, seedling vigor, and to enhance stress tolerance have been widely reported (Rehman et al., 2015; Nawaz et al., 2016; Imran et al., 2017; Petronilio et al., 2021; Lyalina et al., 2023). Meanwhile, rice (Oryza sativa L.) is the world’s second largest source of food and is grown on 163.2 million ha, with an average annual production of 740.9 million tons worldwide (FAOSTAT, 2014). The use of seed priming technique in modern rice production is critical to maintain or increase crop yield because the transition from the traditional manual rice establishment to simplified and mechanized methods require lower seed sowing rate and thus need higher seed vigor (Farooq et al., 2019; Li et al., 2022). The previous studies proved that seed priming was effective in promoting rice seed germination and seedling growth under chilling (Nie et al., 2020; Nie et al., 2022) drought (Zheng et al., 2016), and waterlogging stresses (Hussain et al., 2016). However, the rapid loss of seed viability during storage is a major limitation to the adoption of seed priming in rice production.

Previously, the fast deteriorations of primed-seeds during storage have been observed in rice and other plant species (Sano et al., 2017; Sano and Seo, 2019). Hussain et al. (2015) reported that the germination of primed seeds was significantly reduced after 15 days storage at room temperature. Similar results were also observed in many field crops such as oat, maize and rapeseeds (Wattanakulpakin et al., 2012; Xia et al., 2020; Malek et al., 2022). Meanwhile, the deterioration speed of primed seeds was varied under different storage condition. Wang et al. (2018) reported that increasing the storage temperature did not reduce the viability of primed rice seeds stored under vacuum conditions, whereas seeds stored in the absence of oxygen but with high RH showed reduced longevity. Moreover, the deterioration of primed seeds was suggested to affected by initial seed vigor, the priming-induced seed deterioration was more significant in high vigor seeds, but was much lower in low vigor seeds (Powell et al., 2000; Butler et al., 2009). In addition, the priming methods such as hydropriming, osmoriming and chemical priming were reported to greatly affected the deterioration of primed seeds during storage (Fabrissin et al., 2021). In rice seeds, however, few study has evaluated the differences in deterioration speed of primed rice seeds in response to different priming methods.

The physiological and molecular mechanisms underlying the deterioration of primed seeds have been studied in recent years. A study by Sano et al. (2017) has detected several quantitative trait loci (QTL) that were uniquely associated with primed-seed longevity, which indicated that there might independent mechanisms that controls primed seed deterioration. Several studies suggested the plant hormone signaling may regulate the seed coat permeability and then affected the longevity of primed seeds during storage (Zhou et al., 2020). In addition, the changes of several physiological events including starch metabolism, seed respiration and reactive oxygen species accumulation were suggested to be strongly associated with seed deterioration during storage (Bailly and Kranner, 2011; Jovanović et al., 2014; Wang et al., 2022), and the regulating effects of those metabolic events on seed deterioration and longevity have been well elucidated. Nevertheless, the physiological mechanism underlying the fast deterioration of primed rice seeds need to be further unrevealed.

The present study was conducted to evaluate the differences between two typical priming methods, viz., hydropriming and osmopriming with regards to seed deterioration speed, starch degradation, accumulation and scavenging of reactive oxygen species, and respiratory metabolism by using artificially accelerated aging methods (controlled deterioration test — CDT). The CDT is a classical seed aging method that has widely been used by researchers to study seed longevity within relative short duration by incubating seeds at high temperature and high relative humidity condition (Schwember and Bradford, 2010). The objectives of present study were to evaluate the effects of priming methods on the deterioration speed of rice seeds, and to unravel the physiological mechanisms underlying the differences in seed deterioration between priming methods.

## 2 Materials and methods

### 2.1 Seed source

The rice (*O. sativa* L.) seeds of a local mega variety Chaungliangyoumolizhan (CLYMLZ, hybrid) were used in present study. The initial germination (80.75%, n=4) of the seeds was determined before experiment.

### 2.2 Experimentation

Two experiments were conducted in the laboratory of Hunan Agricultural University, Changsha, China in 2022. In experiment 1 (Exp.1), the rice seeds were subjected to two typical seed priming treatments, viz., hydropriming (HP), osmopriming (PEG). The none-primed seeds (NP) were used as control. After priming treatment, the seeds were aged for 0d (AG0), 2d (AG2), 4d (AG4) and 6d (AG6) by artificial accelerated aging method (Goel et al., 2003). The seeds were then transferred to a growth chamber to conduct standard germination test, and the germination traits and physio-biochemistry attributes of the seeds were determined. In experiment 2 (Exp. 2), the N-acetylcysteine (NAC), which is precursor of glutathione (GSH), one of the most important non-enzymatic antioxidants (Ishibashi et al., 2013), was added to the priming solution to evaluate the effects of reactive oxygen species (ROS) on the deterioration of primed rice seeds. The priming treatments in Exp. 2 include NP, HP, PEG, HP+NAC and PEG+NAC. The primed and non-primed seeds were then aged for 0 and 6d and the germination traits and the accumulation of ROS were determined during germination test. The procedures for seed priming treatment, artificial accelerated aging treatment and standard germination test were described below.

#### 2.2.1 Seed priming treatment

The process of seed priming includes soaking the seeds in priming agents and then dried back to 10% seed moisture content (fresh weight basis) (Hussain et al., 2015). The priming agent used for this study were distilled water (HP), 15% polyethylene glycol (PEG), 0.1 mol L^-1^ N-acetylcysteine dissolved in distilled water (HP+NAC), and 0.1 mol L^-1^ N-acetylcysteine dissolved in 15% polyethylene glycol (PEG+NAC). The seeds were primed in the dark at 25°C for 24h with gentle agitation, the ratio of seed weight to solution volume (w/v) was 1:5. The priming solution was changed after every 12 h. After priming, the seeds were washed with distilled water for 2 min, surface dried by blotting paper and transferred to air dry oven at 25-30°C to reduce the moisture content (Hussain et al., 2015). Dried primed and non-primed seeds were then subjected to different artificial accelerated aging treatments.

#### 2.2.2 Artificial accelerated aging treatment

The methods for artificial accelerated aging treatment were described by Goel et al. (2003) with slight modification. The primed and non-primed rice seeds were placed in nylon bags in an accelerated aging chamber (LH-150S, Zhejiang Tuopu Instrument Co., Ltd. China) with 90 ± 3% air relative humidity (RH) at 45 ± 1°C for 0, 2, 4 and 6d. After aging, the seeds were equilibrated in a chamber with 30 ± 3% RH at 25 ± 1°C for 24h and then vacuumed and sealed in plastic bags and stored at 4°C until use.

#### 2.2.3 Germination test

Fifty healthy seeds from each treatment were evenly placed on two layers of filter paper in petri dishes (14.5 cm diameter). After adding 20 ml water to each treatment, the petri dishes were transferred to growth chamber with 12 h light period, and 30°C (day): 25°C (night temperature). Equal volume of distilled water was applied to all petri dishes when their moisture content decreased. The germination test was last for 7 days. All the experiments were laid out in a completely randomized design with twelve biological replications. Four replications were used to determine seed germination attributes, four replications were sampled at 48 hours after sowing to determine physiological parameters, while the other four replications were sampled at 48 hours to determine oxygen consumption rate.

### 2.3 Measurements

#### 2.3.1 Seed germination

The seed germination was measured daily according to the procedure reported by Crosier et al. (1970). Seed was considered to be germinated when radicle length exceeded 2 mm. Seed germination percentage (%) = No. of germinated seeds/total no. of seeds ×100.

#### 2.3.2 α-amylase activity and soluble sugar content

For the determination of α-amylase activity, 0.3g rice seeds were homogenized and rinsed with 8 ml ice-cold Na-phosphate buffer (pH 7.0, 0.1 M). After centrifuging at 12,000 g for 20 min, the supernatant was collected as a crude extract. Then the α-amylase activity was determined by DNS method (Nie et al., 2022). The total soluble sugar content was determined by phenol sulfuric method as described by Dubois et al. (1956). 0.3 g of rice seeds were oven-dried at 80°C until constant weight and then ground to powder and approximately 100 mg of powder sample was extracted with 5 mL of 80% (v/v) ethanol at 80°C for 30 min; this was repeated three times. After centrifugation, all supernatant was collected in a 100 mL volumetric flask and brought to a final volume of 100 mL by adding distilled water. This solution was used for determination of soluble sugars.

#### 2.3.3 Reactive oxygen species content

The content of superoxide anion (O_2_^-^) and hydrogen peroxide (H_2_O_2_) in imbibed rice seeds was determined by spectrophotometry using Superoxide Determination Kit (BC1295, Cominbio Co. Ltd., China) and Hydrogen Peroxide Determination Kit (BC3595, Cominbio Co. Ltd., China), respectively. All the assays were conducted according to the manufacturer’s instructions. To visualize superoxide anion accumulation in rice seeds, the de-hulled seeds was imbibed in 0.1% nitro-blue tetrazolium (NBT) solution for 3 hr at room temperature under dark, the unbound dye was washed and removed using 100% ethanol, after then the images were taken under a bright field (Kumari et al., 2021).

#### 2.3.4 Lipid peroxidation and antioxidant enzyme activity

To determine the lipid peroxidation and antioxidant enzymes activity, 0.2 g fresh samples were ground in 1.5 ml of 50 mM cool phosphate buffer [pH 7.0, containing 1% (w/v) PVP]. The homogenate was centrifuged at 15,000g for 20 min at 4 °C, the supernatant was then used for further assays. The lipid peroxidation level was determined in terms of malondialdehyde (MDA) content by measuring the absorbance at 532nm and 600nm according to the method of Dhindsa et al. (1981), the MDA content was calculated as described by Heath and Packer (1968). To evaluate the changes in antioxidant system in primed rice seeds during aging, the activity of three dominant antioxidant enzymes, viz. superoxide dismutase (SOD), peroxidase (POD) and catalase (CAT) was determined by spectrophotometry as described by Wang et al. (2018).

#### 2.3.5 Oxygen consumption rate

The oxygen consumption of rice seeds was measured by an optic oxygen microsensor (OXY1-ST, Presens Gmbh, Germany). twenty rice seeds were put into 2 ml glassy autosampler vials (Agilent, America). The vial was then equilibrated in a growth chamber (dark, 30°C) for 15 minutes with the vial tightly sealed to prevent air diffusion. After then, the needle type microsensor (PSt7) was inserted to the vial to monitor oxygen concentration.

#### 2.3.6 ATP content and malate dehydrogenase activity

The ATP content was determined by the method described by Strehler and Totter (1952). The enzyme activity of malate dehydrogenase (MDH) was analyzed by monitoring the attenuation rate of NADH at 340 nm as described by Nie et al. (2020).

### 2.4 Statistical analysis

All data from present experiment were expressed as the mean value of four biological replications. Statistix11.0 was used to analyze the data. We used one-way ANOVA, with seed priming treatments and no-priming control as grouping factors, morphological and physiological indicators (as mentioned above) as response variables. The differences were analyzed using the Least Significance Difference (LSD) test at the 0.05 probability level. The figure illustration was performed using SigmaPlot 12.5.

## 3 Results

### 3.1 Seed germination ability of primed and non-primed rice seeds after accelerated aging

**Figure 1:**
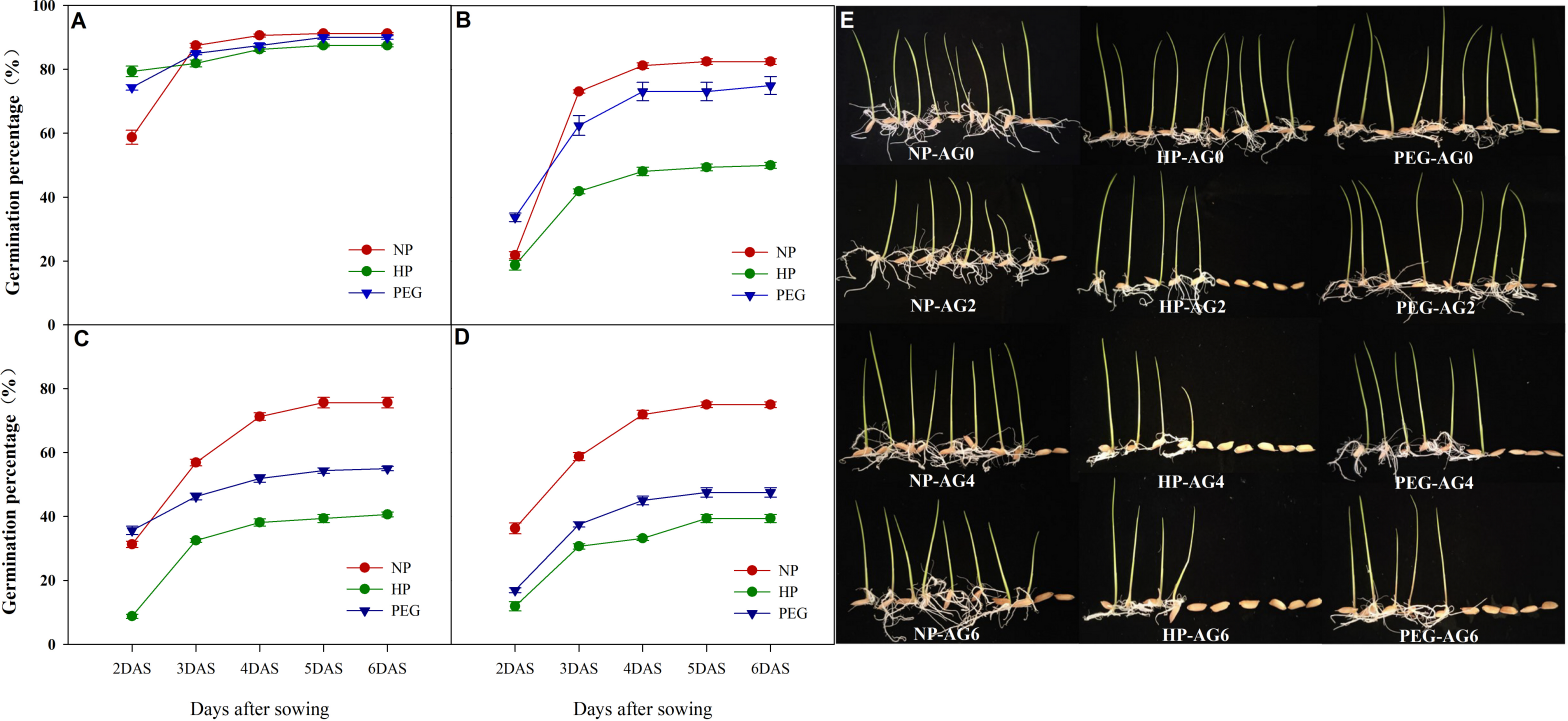
Germination dynamics and seedling growth of hydro-primed, osmo-primed and non-primed seeds after 0, 2, 4 and 6 days of accelerated aging (Exp.1). **(A)** accelerated aging for 0d. **(B)** accelerated aging for 2d. **(C)** accelerated aging for 4d. **(D)** accelerated aging for 6d. **(E)** the seedling growth after 7 days of germination. NP, no priming control; HP, hydropriming; PEG, osmopriming; AG0, 2, 4, 6, accelerated aging for 0, 2, 4 and 6 days; DAS, days after sowing. Seed germination data was recorded from 2 DAS till constant at 6 DAS. Error bars indicate standard error (n=4).

The germination percentage of primed and non-primed rice seeds after 0, 2, 4 and 6 days of accelerated aging treatments were shown in **Figure 1**. At AG0, both seed priming treatments increased the germination speed than the non-primed control, which was increased by 35.1% and 26.6% for HP and PEG treatments, and the germination index and vigor index of rice seeds under both priming treatments were significantly improved (data not shown). However, the final germination percentage did not differ between primed and non-primed seeds. Meanwhile, the accelerated seed aging for two days greatly reduced the germination speed and final germination percentage of both primed and non-primed seeds, but the primed seeds exhibited much weaker aging tolerance than non-primed seeds, as the final germination percentage was decreased by 50.8% and 9.1% for HP and PEG respectively as compared with CK. Extending the accelerated aging period further reduced the germination characteristics of primed rice seeds. When averaged across priming treatments, the germination percentage of primed seeds was decreased by 36.7% and 42.0% at AG4 and AG6, respectively as compared with that of CK, suggesting that the primed seeds deteriorated more easily than non-primed seeds. In addition, significant differences in deterioration speed were observed between HP and PEG treatments. The germination speed (germination percentage at 2 DAS) of PEG-primed seeds was higher than that of CK and HP from AG2 to AG4, and the significant reduction was observed only up to AG6. On the other hand, the germination speed of HP-primed seeds was significantly decreased by 33.3%, 26.1% and 17.1% at AG2, AG4 and AG6, respectively, as compared to PEG treatment. Similarly, HP showed much higher decrease in final germination percentage than PEG during accelerated aging.

### 3.2 α-amylase activity and total soluble sugar content of primed and non-primed rice seeds after accelerated aging

**Figure 2:**
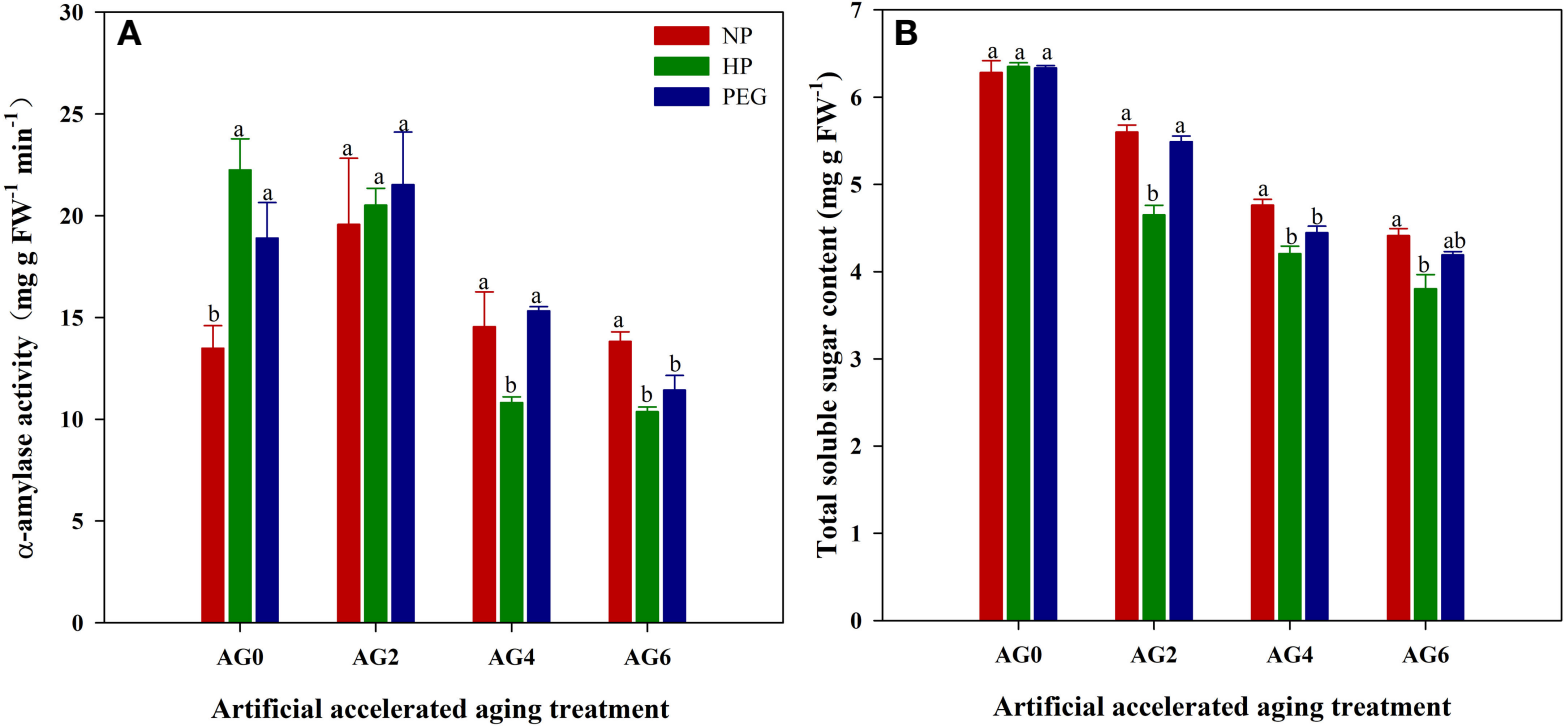
α-amylase activity and total soluble sugar content of hydro-primed, osmo-primed and non-primed seeds after 0, 2, 4 and 6 days of accelerated aging (Exp.1). **(A)** α-amylase activity. **(B)** total soluble sugar content. NP, no priming control; HP, hydropriming; PEG, osmopriming; AG0, 2, 4, 6, accelerated aging for 0, 2, 4 and 6 days. Error bars indicate standard error (n=4). Different lowercase letters denote statistical differences at the 5% level according to LSD test among seed treatments within the same accelerated aging time.

Seed priming significantly increased the α-amylase activity in rice seeds, which was increased by 64.9% and 40.1% for HP and PEG, respectively as compared with CK (**Figure 2**). Similarly, a slight increase in total soluble sugar content was also observed in primed rice seeds, suggesting that the starch degradation ability of rice seeds could be improved by seed priming. However, the benefits of seed priming in promoting starch degradation were offset by accelerated aging, as the α-amylase activity and total soluble sugar content in primed rice seeds were lower or showed no difference from that in CK at AG2, and were greatly reduced from AG4 to AG6. Similar to the changes in seed germination characteristics, the reduction in α-amylase activity and total soluble sugar content during accelerated aging was greater in HP-primed seeds than in PEG-primed seeds. At AG2, although the α-amylase activity did not differ between the two priming treatments, the total soluble sugar content was reduced by 15.4% in HP-primed seeds than in PEG-primed seeds. Furthermore, the α-amylase activity in HP-primed seeds was reduced by 29.4% and 9.2% at AG4 and AG6 respectively, compared to that in PEG-primed seeds, and the reduction in total soluble sugar content was 5.3% and 9.2% at AG4 and AG6, respectively.

### 3.3 ROS accumulation, antioxidant enzyme activity and malondialdehyde content of primed and non-primed rice seeds after accelerated aging

**Figure 3:**
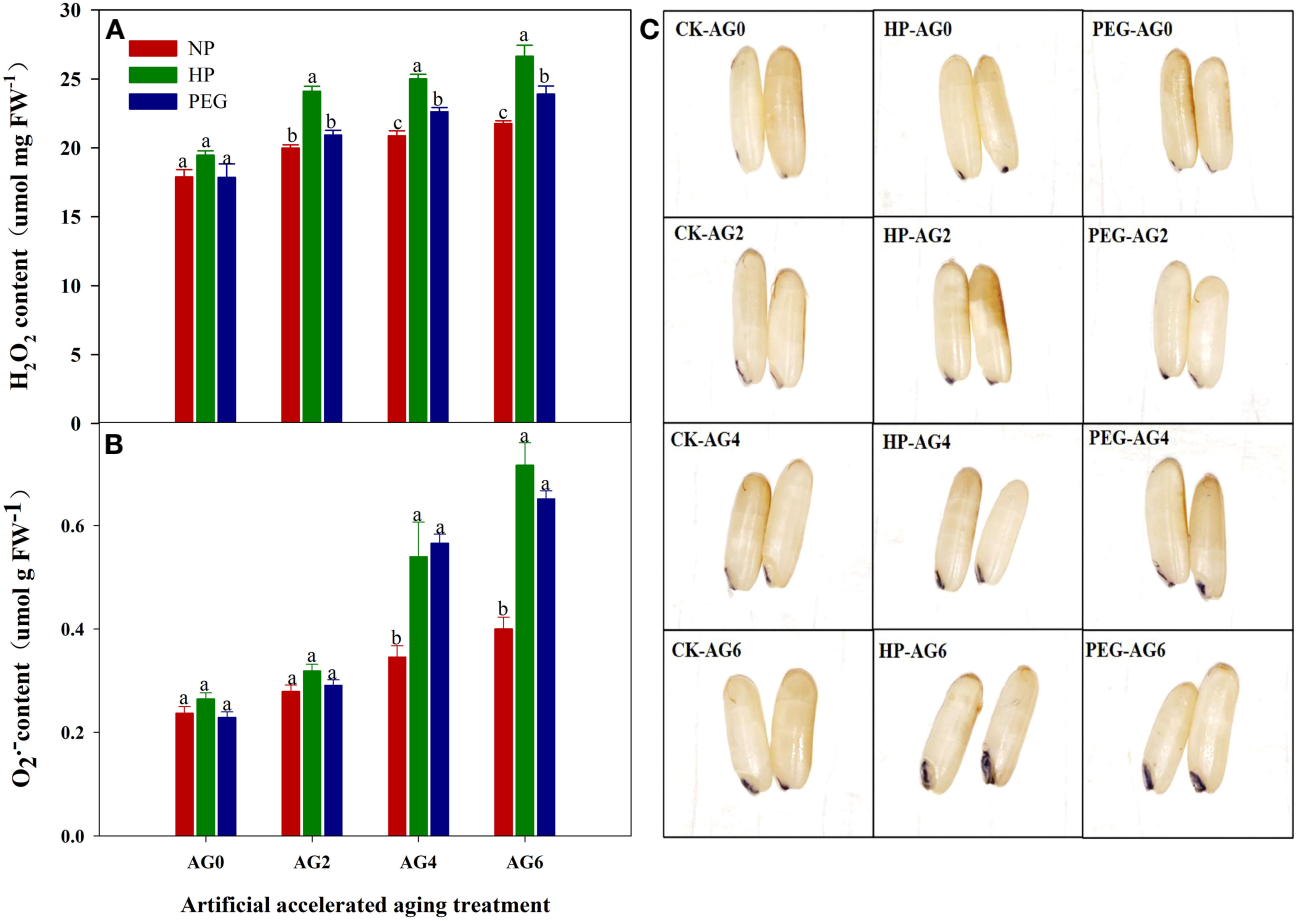
superoxide anion, hydrogen peroxide content and NBT staining of hydro-primed, osmo-primed and non-primed seeds after 0, 2, 4 and 6 days of accelerated aging (Exp.1). **(A)** hydrogen peroxide content **(B)** superoxide anion content. **(C)** NBT staining. NP, no priming control; HP, hydropriming; PEG, osmopriming; AG0, 2, 4, 6, accelerated aging for 0, 2, 4 and 6 days; O_2_^-^, superoxide anion; H_2_O_2_, hydrogen peroxide Error bars indicate standard error (n=4). Different lowercase letters denote statistical differences at the 5% level according to LSD test among seed treatments within the same accelerated aging time.

**Figure 4:**
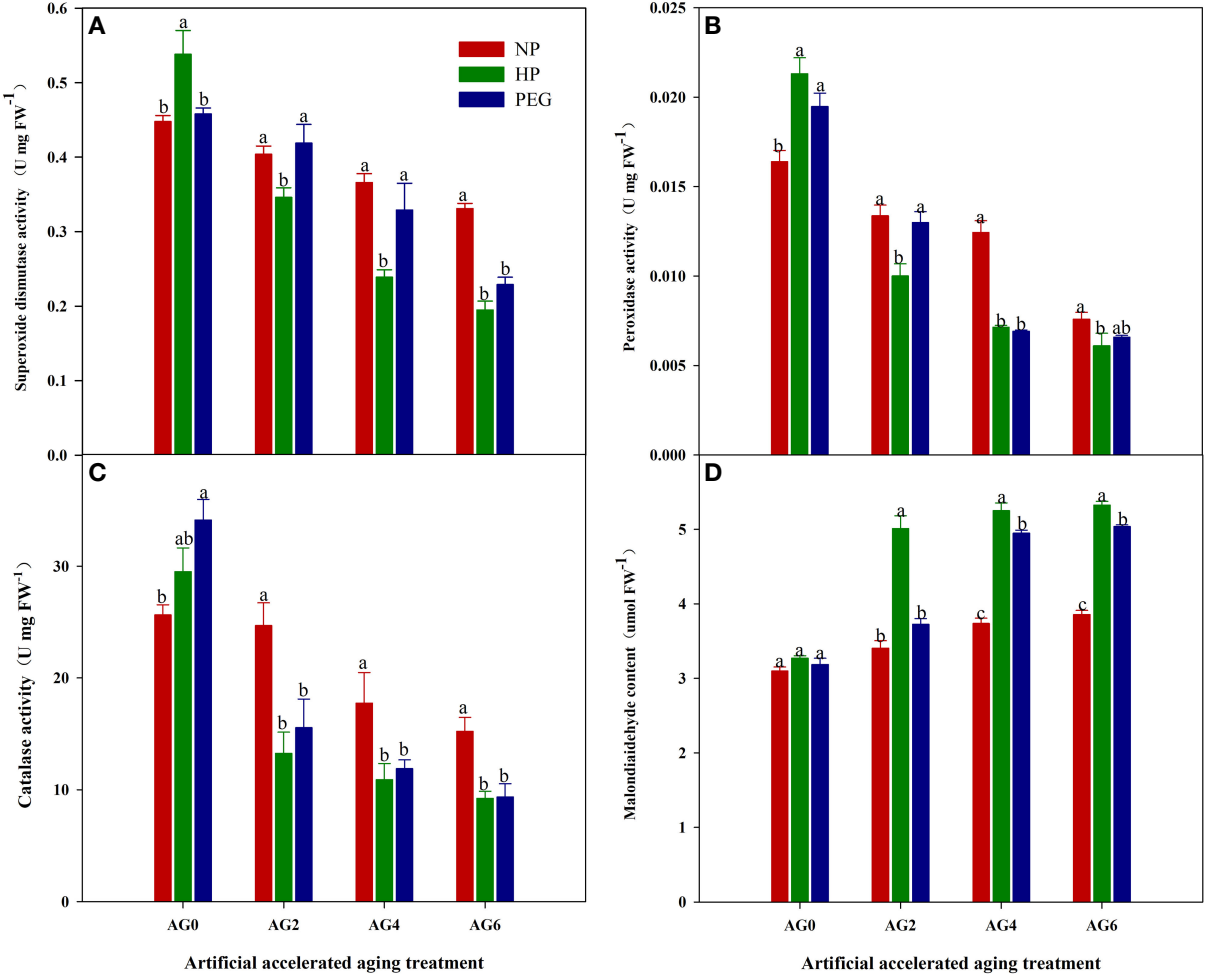
superoxide dismutase, peroxidase, catalase activity and malondialdehyde content of hydro-primed, osmo-primed and non-primed seeds after 0, 2, 4 and 6 days of accelerated aging (Exp.1). **(A)** superoxide dismutase activity. **(B)** peroxidase activity. **(C)** catalase activity. **(D)** malondialdehyde content. NP, no priming control; HP, hydropriming; PEG, osmopriming; AG 0, 2, 4, 6, accelerated aging for 0, 2, 4 and 6 days. Error bars indicate standard error (n=4). Different lowercase letters denote statistical differences at the 5% level according to LSD test among seed treatments within the same accelerated aging time.

The primed and non-primed rice seeds showed similar ROS accumulation and lipid peroxidation level before accelerated aging, as the content of superoxide anion (O_2_^-^), hydrogen peroxide (H_2_O_2_) and malondialdehyde (MDA) in both HP- and PEG-primed seeds did not show significant difference compared with that in CK at AG0 (**Figures 3**, **4D**), but the activity of antioxidant enzymes in primed rice seeds was significantly increased (**Figures 4A–C**). The HP-primed seeds exhibited faster and higher ROS accumulation and lipid peroxidation than the PEG-primed seeds. At AG2, O_2_^-^, H_2_O_2_ and MDA levels in PEG-primed seeds showed no difference from those in CK, but H_2_O_2_ and MDA levels in HP-primed seeds was increased by 15.3% and 34.4%, respectively as compared with those in CK. Although the ROS accumulation in PEG-primed seeds was progressively increased when the accelerated aging period was extended to 4-6 days, significantly lower O_2_^-^ and MDA contents were observed in PEG-primed seeds than in HP-primed seeds. In contrast, the activity of antioxidant enzymes was greatly reduced after accelerated aging, and the reduction was more pronounced in HP-primed seeds than in PEG-primed seeds, as the superoxide dismutase (SOD) activity of PEG-primed seeds showed no significant difference with CK, but was 21.1% and 37.7% higher than that of HP-primed seeds at AG2 and AG4, respectively. Similarly, the peroxidase (POD) activity in PEG-primed seeds showed no difference with CK but was significantly higher than HP at AG2. However, catalase (CAT) activity was significantly reduced under both priming treatments compared to CK and no difference was found between HP and PEG after accelerated aging.

### 3.4 Effects of NAC on the germination ability and ROS accumulation of primed rice seeds after accelerated aging

**Figure 5:**
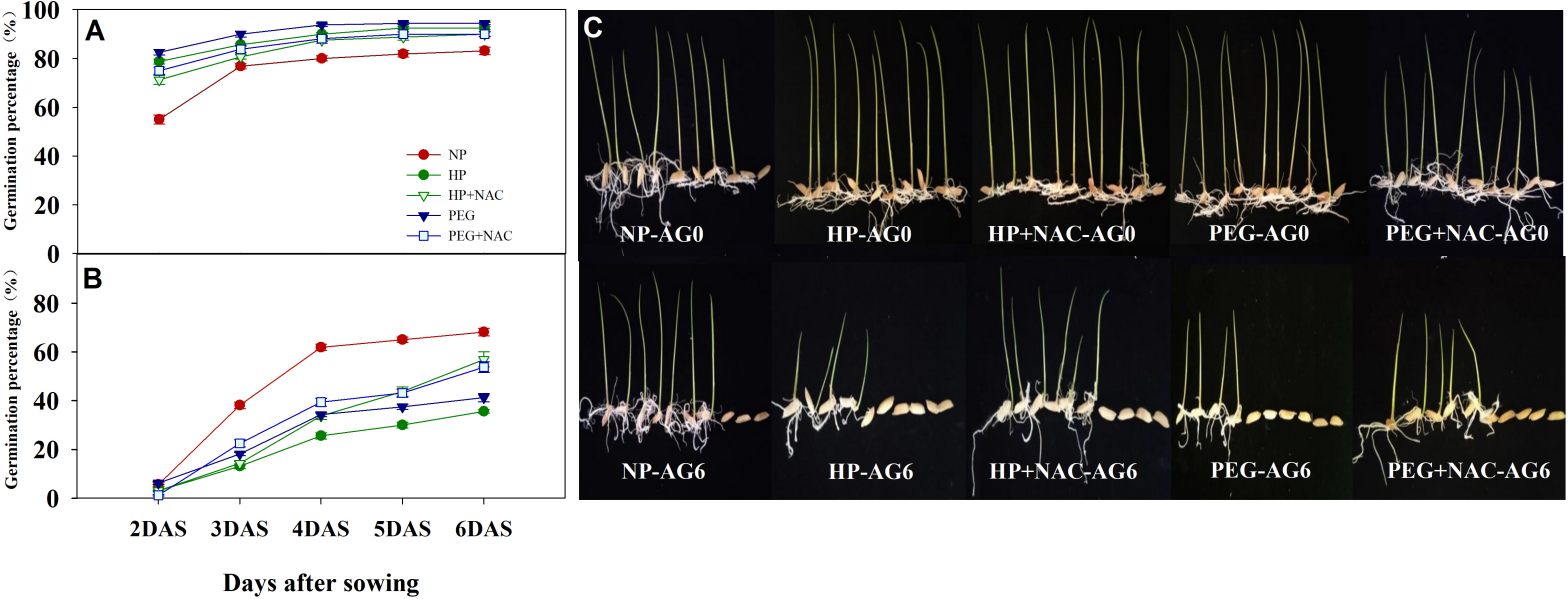
Effects of N-acetylcysteine on the germination and seedling growth of hydro-primed, osmo-primed seeds after 0 and 6 days of accelerated aging (Exp.2). **(A)** accelerated aging for 0d. **(B)** accelerated aging for 6d. **(C)** the seedling growth after 7 days of germination. NP, no priming control; HP, hydropriming; PEG, osmopriming; NAC, N-acetylcysteine; DAS, days after sowing; AG 0 and AG6, accelerated aging for 0 and 6 days. Error bars indicate standard error (n=4).

**Figure 6:**
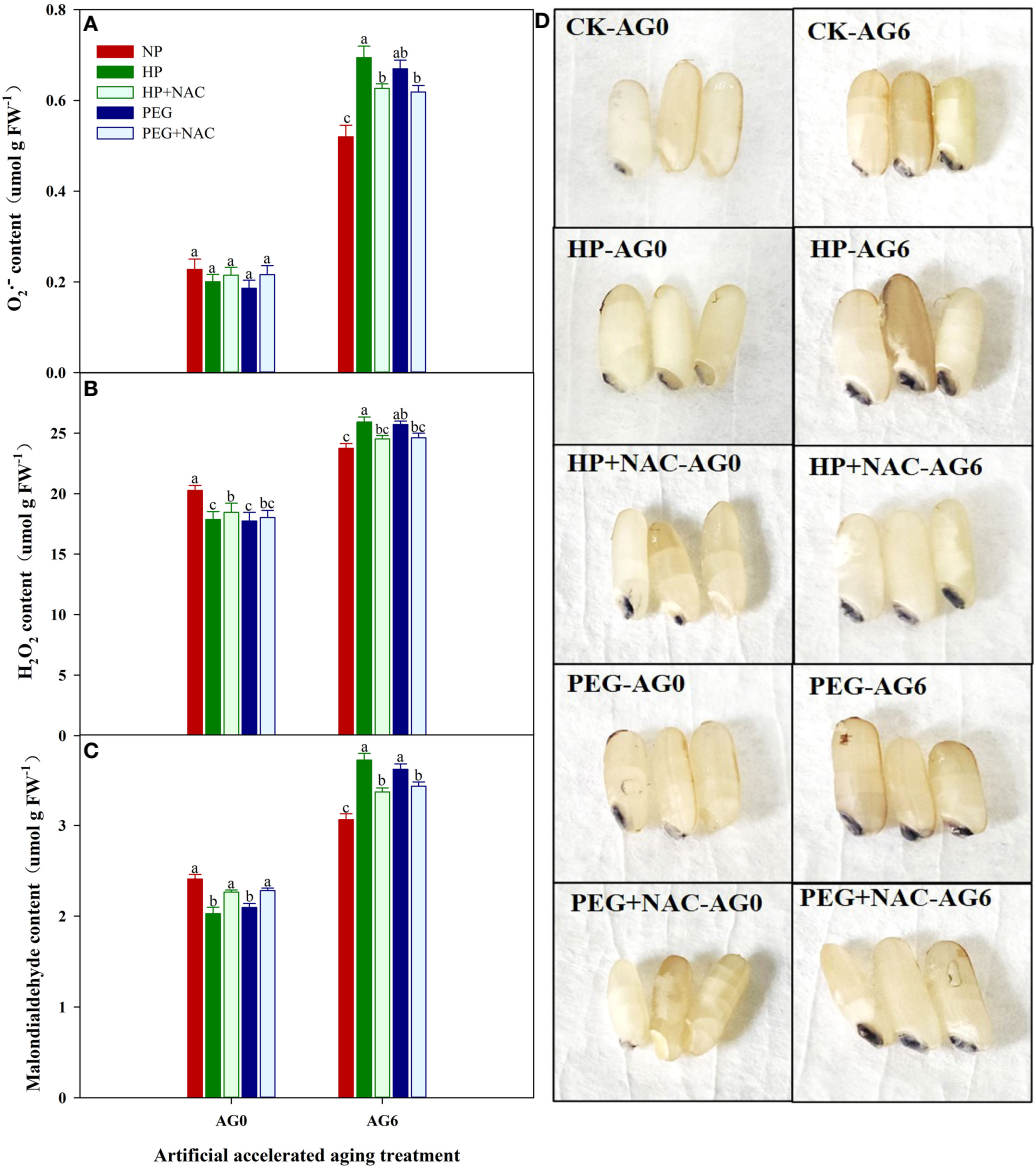
Effects of N-acetylcysteine on the reactive oxygen accumulation and lipid peroxidation of hydro-primed, osmo-primed seeds after 0 and 6 days of accelerated aging (Exp.2). **(A)** hydrogen peroxide content. **(B)** superoxide anion content. **(C)** malondialdehyde content. **(D)** NBT staining. NP, no priming control; HP, hydropriming; PEG, osmopriming; NAC, N-acetylcysteine; AG 0 and AG6, accelerated aging for 0 and 6 days. Error bars indicate standard error (n=4). Different lowercase letters denote statistical differences at the 5% level according to LSD test among seed treatments within the same accelerated aging time.

The use of NAC during priming greatly affected the germination performance of primed rice seeds after accelerated aging (**Figures 5**, **6**). At AG0, the germination speed and final germination percentage of both HP+NAC and PEG+NAC treatments were significantly higher than that of CK, but relatively lower than that of HP and PEG treatments. Although all the priming treatments exhibited significantly lower germination percentage than that of CK after accelerated aging, the use of NAC greatly inhibited the deterioration of primed rice seeds, as HP+NAC and PEG+NAC treatments increased the final germination percentage by 59.6% and 50.8%, respectively as compared with HP, and those were increased by 37.8% and 30.3%, respectively as compared with PEG (**Figure 5**). Meanwhile, the ROS accumulation and lipid peroxidation in primed seeds were significantly reduced by NAC after accelerated aging (**Figure 6**). When compared with HP-primed seeds, the O_2_^-^, H_2_O_2_ and MDA content in HP+NAC-primed seeds were reduced by 9.8%, 5.4% and 9.4%, respectively. Similarly, The PEG+NAC treatment decreased the O_2_^-^, H_2_O_2_ and MDA content by 7.7%, 4.3% and 5.2%, respectively as compared with PEG.

### 3.5 Oxygen consumption rate of primed and non-primed rice seeds after accelerated aging

**Figure 7:**
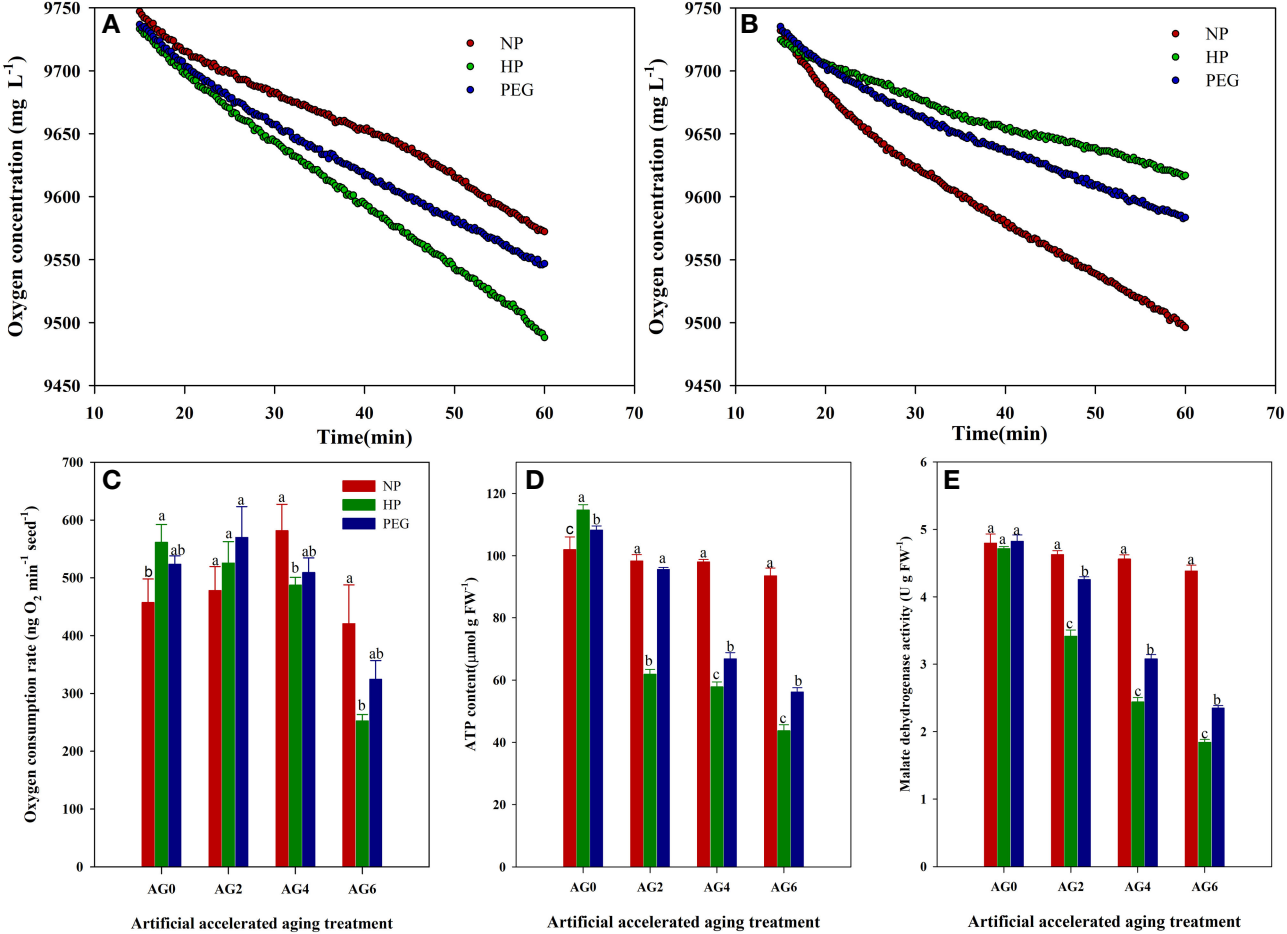
oxygen consumption rate, ATP content and malate dehydrogenase activity of hydro-primed, osmo-primed and non-primed seeds after 0, 2, 4 and 6 days of accelerated aging (Exp.1). **(A)** changes in oxygen concentration after 0 days of accelerated aging. **(B)** changes in oxygen concentration after 6 days of accelerated aging. **(C)** oxygen consumption rate. **(D)** ATP content. **(E)** malate dehydrogenase activity.NP, no priming control; HP, hydropriming; PEG, osmopriming; AG 0, 2, 4, 6, accelerated aging for 0, 2, 4 and 6 days. Error bars indicate standard error (n=4). Different lowercase letters denote statistical differences at the 5% level according to LSD test among seed treatments within the same accelerated aging time.

The oxygen consumption rate and the respiration-related indicators of primed and non-primed rice seeds were shown in **Figure 7**. At AG0, both HP and PEG priming increased the oxygen consumption rate and the ATP content as compared with CK, suggesting that the respiratory metabolism of rice seeds could be improved by seed priming treatments. However, the activity of malate dehydrogenase (MDH), which is the key enzyme involved in the tricarboxylic acid cycle, did not vary among treatments. The primed seeds exhibited relatively higher oxygen consumption rate than non-primed rice seeds at AG2, but extending aging period to 4-6 days significantly decreased the oxygen consumption in primed rice seeds as compared to that in non-primed seeds. Similarly, the ATP content and MDH activity in primed seeds were progressively decreased after accelerated aging. In addition, significant variations in respiratory activity were observed between HP and PEG treatments before and after accelerated aging. Compared with PEG, HP-primed seeds exhibited 7.3% and 6.0% higher oxygen consumption rate and ATP content, respectively, at AG0. In contrast, both oxygen consumption rate, ATP content and MDH activity in HP-primed seeds were drastically decreased after accelerated aging, and the decrease trend was significantly higher than that of PEG-primed seeds. For example, the ATP content in HP-primed seeds was decreased by 35.2%, 13.5% and 22.2% at AG2, AG4 and AG6, respectively as compared with that in PEG-primed seeds.

## 4 Discussion

The effects of seed priming on seed storability have been assessed in multiple crop species and the negative effects have been observed in most cases. However, little attention has been given to the difference in deterioration speed of primed seeds in a controlled and constant environment, which might be affected by cultivars and priming methods. In the present study, it was observed that PEG-primed rice seeds exhibited lower deterioration speed than hydro-primed rice seeds under controlled accelerated aging condition in terms of germination percentage and germination speed, These results suggest that manipulating the priming methods can potentially extend the storability of primed rice seeds. Previously, several studies have observed the regulatory effects of priming type, imbibition time, temperature and drying speed on seed longevity, which have been summarized and reviewed by Fabrissin et al. (2021). However, conflicting results exist between different studies. For instance, PEG-priming reduced the longevity of leek seeds (Dearman et al., 1987), but extended the storability of onion seeds (Dearman et al., 1986). The inconsistent findings may be attributed to variations in aging methods, priming protocols, and cultivars selections employed across various investigations. In rice seeds, Wang et al. (2018) found that the deterioration speed of hydro-primed seeds showed no difference with that of PEG-primed seeds under vacuum or low-temperature condition, but deteriorated faster under room temperature and 60%RH (Wang et al., 2018). This finding is consistent with that of Moradi and Younesi (2009) and with the present work that conducted under accelerated aging conditions.

Seed hydration is a crucial step in seed priming that affects the germination performance and storage of seeds after priming. It has been reported that prolonged hydration during priming had a deleterious effect on the subsequent germination of seeds after storage (Fabrissin et al., 2021). This is likely because prolonged hydration activated various metabolic events that are essential for seed germination (Bewley, 1997; Lemmens et al., 2019), but can be harmful for seed storage. In the present study, the seed hydration duration was consistent between the two priming methods. However, the water absorption rate was lower in PEG-priming compared to hydro-priming (unpublished data; Al-Karaki, 1998), which could have led to varied seed hydration levels and consequently different deterioration rate between the two priming methods. To further explore why hydro-primed seeds deteriorated faster than PEG-primed seeds, several indicators that related to the key metabolic events during seed deterioration or germination including starch degradation, respiration and ROS scavenging were determined. The results suggested that the starch degradation ability was highly correlated with the deterioration of primed rice seeds. Specifically, the α-amylase activity and total soluble sugar content in hydro-primed seeds decreased faster than those in PEG-primed seeds. The reduced α-amylase activity and total soluble sugar content in primed rice seeds during storage was also observed by Hussain et al. (2015). It was suggested that starch metabolism is the major driving force for rice seed germination and could support plants to grow faster under various environment (Murata et al., 1968). The starch degradation ability during rice seed germination is strongly associated with respiratory metabolism (Wang et al., 2016). The activation of α-amylase enzymes triggered an increase in respiration rate (Paul and Mukherji, 1972), while seed respiration provides essential energy for starch degradation and subsequent seed germination (Nie et al., 2020). These findings indicated that the decreased seed respiration observed in the present study may contribute to the reduced starch degradation ability in aged primed seeds. Moreover, the decline in starch degradation ability in deteriorated seeds may be related to the oxidative stress of seeds during storage (Wang et al., 2018). Membrane damage and solute leakage triggered by lipid peroxidation could inevitably impair starch metabolism that occurred in seeds embryo and starchy endosperms (Riewe et al., 2017; Wiebach et al., 2020), which led to a decrease in seed viability. The present study also found that the ROS accumulation and lipid peroxidation may be the key events that contribute to different deterioration speed of primed rice seeds. Although the H_2_O_2_ and O_2_^-^ levels did not differ between hydro-primed seeds and PEG-primed seeds before accelerated aging, the improved antioxidant enzyme activity may indicate higher ROS generation level in hydro-primed rice seeds than in PEG-primed and non-primed ones. During storage, the antioxidant enzymes generally losses their function because the mobilization and diffusion of the reactive substrate are strictly limited in the glassy cytoplasm of dry seeds (Kranner et al., 2010; Kumar et al., 2015). However, the seed respiration accompanied with ROS production will not stop during storage, which ultimately lead to lipid peroxidation and seed death during storage (Zhang et al., 2021). In present study, we hypothesize that the faster deterioration of hydro-primed rice seeds might be attributed to faster ROS production and accumulation during accelerated aging, because the hydro-primed seeds exhibited significantly higher H_2_O_2_ and MDA content than PEG-primed seeds, and the antioxidant enzyme activity were greatly inhibited. This hypothesis might be partially proved by the NAC addition experiment, in which the seed deterioration, as well as the ROS accumulation and lipid peroxidation of hydro-primed seeds after aging was greatly inhibited by NAC. NAC is an important factor, as well as other nonenzymatic antioxidants, due to the fact that they have lower molecular masses, having greater mobility and quenching ability in regard to enzymatic antioxidants (Gerna et al., 2022). Nevertheless, the NAC content in primed rice seeds was not determined in present study, and the possible function of NAC that regulate the deterioration speed of primed rice seeds need to be further evaluated.

In the present study, elevated ROS levels in hydro-primed rice seeds might be attributed to differences in seed physiological status influenced by seed hydration. In the priming process, seeds are allowed to absorb water up to phase II of the three phases water absorption model (Bradford, 2017), during which several metabolic events that favors seed germination would be activated (Bradford, 2017). The research of Nie et al. (2020) observed the repair and biogenesis of mitochondria during priming. This restoration of mitochondrial function is accompanied by a rapid increase in ROS production (Farooq et al., 2021). Previous research has also shown a negative correlation between seed respiration and seed storability (Ferguson et al., 1990; Vertucci and Roos, 1990). In the present study, hydropriming resulted in increased seed oxygen consumption and ATP levels prior to aging. This effect might be attributed to the faster water absorption rate of hydro-priming compared to PEG-priming (Al-Karaki, 1998), which may trigger higher respiratory activity during a specific priming duration (24h in present study), and subsequently lead to accelerated ROS accumulation and lipid peroxidation. However, the present study only determined the respiration rate, ATP content and MDH activity, the mechanisms underlying why hydro-primed seeds exhibit higher respiration rate after priming remained to be unknown, the changes in respiratory metabolism, and their possible relationship with the deterioration of primed rice seeds needs to be explored in the future.

## 5 Conclusion

The hydroprimed rice seeds exhibited significantly faster deterioration speed than that of PEG-primed seeds in terms of germination speed and percentage. Additionally, α-amylase activity and total soluble sugar content in hydroprimed seeds reduced by 19.3% and 10.0% respectively after aging compared to PEG-primed seeds. Such effects were observed to be significantly associated with the elevated ROS accumulation and lipids peroxidation. The levels of superoxide anion, hydrogen peroxide, and malondialdehyde in hydroprimed seeds were 4.4%, 12.3%, and 13.7% higher than those in PEG-primed seeds after aging. This effect could be attributed to the augmented respiratory metabolism in hydroprimed seeds. Additionally, the simultaneous use of N-acetylcysteine with HP and PEG priming significantly inhibited the deterioration of primed rice seeds. These findings imply that the ability to eliminate reactive oxygen species may be the critical factor affecting the deterioration speed of primed rice seeds during storage. Furthermore, this study demonstrates the potential to lengthen the storage duration of primed rice seeds by adjusting the priming methods. However, the physiological and molecular mechanisms underlying why hydro-primed seeds exhibit a higher respiration rate and faster ROS accumulation than PEG-primed seeds remain unknown and require future exploration.

## Data availability statement

The original contributions presented in the study are included in the article/supplementary files, further inquiries can be directed to the corresponding author.

## Author contributions

MR: Data curation, Formal Analysis, Investigation, Methodology, Writing – original draft. BT: Investigation, Writing – original draft. JX: Investigation, Writing – original draft. ZY: Investigation, Writing – original draft. HZ: Writing – review & editing. QT: Funding acquisition, Writing – review & editing. XZ: Funding acquisition, Writing – review & editing. WW: Conceptualization, Funding acquisition, Writing – original draft.
